# Advances in cardiovascular risk assessment indicators in obstructive sleep apnea

**DOI:** 10.3389/fpubh.2026.1878113

**Published:** 2026-08-05

**Authors:** Tong Feng, Yuanyuan Liu, Yubing Yue, Chunfang Zeng, Yinhe Feng

**Affiliations:** Department of Respiratory and Critical Care Medicine, Deyang People's Hospital, Deyang, China

**Keywords:** biomarkers, cardiovascular risk assessment, hypoxic burden, obstructive sleep apnea, precision medicine

## Abstract

Obstructive sleep apnea (OSA) is a highly prevalent sleep-related breathing disorder that is closely associated with cardiovascular morbidity and mortality. Although the apnea–hypopnea index (AHI) remains the conventional metric for diagnosing and grading OSA severity, it inadequately reflects the heterogeneous pathophysiological burden of the disease and has limited predictive value for cardiovascular outcomes. This review summarizes recent advances in cardiovascular risk assessment indicators in OSA, with a focus on mechanisms of OSA-related cardiovascular injury, limitations of traditional metrics, emerging physiological indices, circulating biomarkers, phenotype-based stratification, and risk evaluation in patients with specific cardiovascular comorbidities. Intermittent hypoxia, sleep fragmentation, and intrathoracic pressure swings jointly contribute to sympathetic activation, oxidative stress, systemic inflammation, endothelial dysfunction, hypercoagulability, metabolic dysregulation, and cardiac remodeling. Novel physiological indicators, including hypoxic burden, heart rate response, pulse wave amplitude drop index, and respiratory effort–related metrics, may better quantify OSA-related cardiovascular stress than AHI alone. In parallel, biomarkers reflecting inflammation, endothelial dysfunction, coagulation activation, myocardial injury, and microRNA-mediated regulation show potential for identifying high-risk subgroups. Phenotype-based approaches, such as classifications integrating symptoms, hypoxic patterns, and cardiovascular risk, provide a promising framework for precision management. Future research should standardize these novel indicators, validate their associations with hard cardiovascular outcomes, and develop multimodal prediction models to guide individualized therapeutic strategies.

## 1 Introduction

Obstructive sleep apnea (OSA) is a common sleep-related breathing disorder characterized by recurrent complete or partial collapse of the upper airway during sleep. Globally, OSA affects nearly one billion individuals, and its prevalence has increased significantly due to the widespread occurrence of obesity, physical inactivity, and diabetes (1). The association between OSA and cardiovascular disease has been extensively confirmed by epidemiological studies. Observational studies indicate that OSA is an independent risk factor for hypertension, poor blood pressure control, stroke, myocardial infarction, heart failure, arrhythmias, sudden cardiac death, and all-cause mortality (2). For instance, among patients with acute coronary syndrome, the prevalence of mild OSA is extremely high, reaching up to 70% as measured by polysomnography (3). However, despite clear pathophysiological links and supportive observational data, large randomized controlled trials have not consistently demonstrated that continuous positive airway pressure (CPAP) therapy significantly improves major cardiovascular outcomes (2, 4). Possible explanations for these “negative” results include limitations in trial design, low adherence to CPAP therapy, and the failure to adequately account for the highly heterogeneous nature of OSA (2).

**Figure 1:**
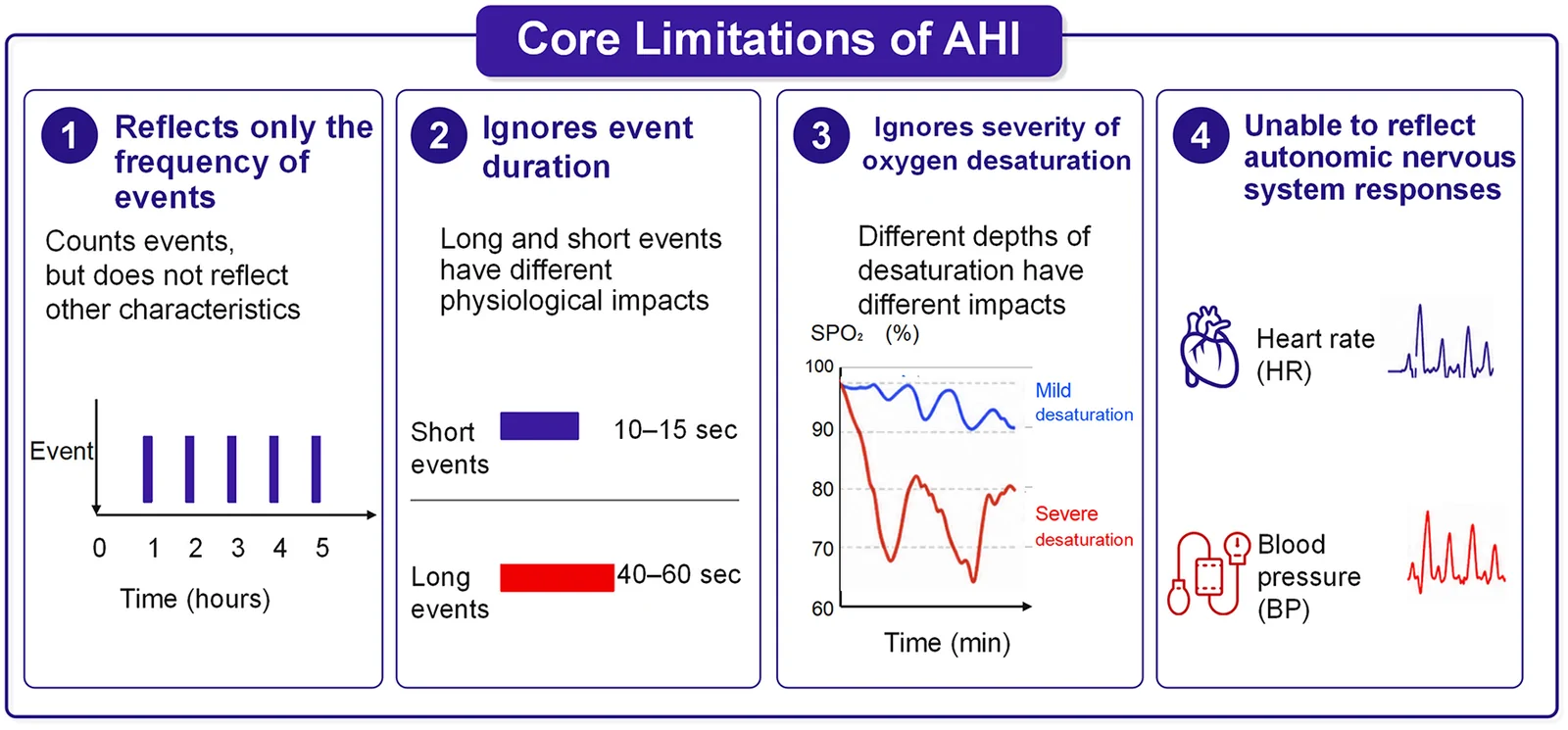
Limitations of AHI: why it fails to adequately predict cardiovascular risk.

Traditionally, the diagnosis and severity assessment of OSA have relied primarily on the apnea–hypopnea index (AHI). However, as a single frequency-based metric, AHI fails to capture the intrinsic pathophysiological complexity of the disease, such as the depth and duration of hypoxemia, autonomic nervous responses, and the consequent systemic effects (5) (Figure 1). It also cannot effectively differentiate between distinct clinical phenotypes or predict an individual patient's risk of cardiovascular events and response to therapy (5). These limitations have resulted in significant shortcomings in using AHI to guide treatment decisions and assess cardiovascular risk in both clinical practice and research. Consequently, in the era of precision medicine, there is an urgent research and clinical need to identify novel indicators that more accurately reflect OSA-related cardiovascular stress, enable risk stratification, and predict therapeutic benefit (2, 6). This review aims to summarize the research progress on cardiovascular risk assessment indicators in OSA, focusing on core pathophysiological mechanisms, covering the limitations of traditional metrics, novel physiological indices, biomarkers, integrated phenotyping strategies, and risk evaluation in the context of specific comorbidities, while also discussing clinical implementation challenges and future directions.

## 2 Core pathophysiological mechanisms of OSA-related cardiovascular injury

The mechanisms by which OSA induces cardiovascular injury are complex and interrelated, with core pathways arising from three characteristic nocturnal events: intermittent hypoxia, sleep fragmentation, and dramatic fluctuations in intrathoracic pressure (7, 8). These events collectively activate multiple downstream pathways, ultimately promoting the onset and progression of cardiovascular disease.

Intermittent hypoxia (IH) is the most distinctive pathophysiological feature of OSA, characterized by recurrent cycles of hypoxia and reoxygenation. This unique pattern of hypoxia, rather than sustained hypoxia, is the critical trigger for systemic injury (9, 10). IH induces oxidative stress and systemic inflammatory responses through activation of hypoxia-inducible factors (11). Studies have confirmed that patients with OSA exhibit elevated levels of inflammatory markers such as C-reactive protein, tumor necrosis factor-alpha, and interleukin-6 (12, 13). IH and oxidative stress jointly impair vascular endothelial function, leading to decreased bioavailability of nitric oxide and increased release of endothelin-1, which in turn causes vasoconstriction, smooth muscle cell proliferation, and atherosclerosis (14, 15). A meta-analysis of individual patient data showed that, among adults without overt cardiovascular disease, severe OSA was independently associated with an increased risk of endothelial dysfunction (16). In addition, IH activates the sympathetic nervous system, resulting in persistently elevated catecholamine levels, which lead to increased heart rate, heightened vascular resistance, and elevated blood pressure (1, 17, 18).

Sleep fragmentation, caused by recurrent micro-arousals, can independently trigger sympathetic overactivation and impaired baroreflex function, even in the absence of significant hypoxia. Disruption of sleep architecture also affects the hypothalamic–pituitary–adrenal axis, exacerbating metabolic dysregulation. Dramatic fluctuations in intrathoracic pressure, which can reach up to −80 cmH_2_O, directly increase cardiac load. Inspiratory efforts against an obstructed airway generate substantial negative intrathoracic pressure, which simultaneously raises left ventricular transmural pressure (afterload) and increases venous return to the heart (preload), resulting in elevated mechanical stress and wall tension; over time, this can promote myocardial remodeling and heart failure (7).

These core mechanisms converge to promote hypertension, atherosclerosis, cardiac remodeling, and arrhythmias through pathways involving sympathetic activation, oxidative stress, inflammation, endothelial dysfunction, hypercoagulability, and metabolic disturbances (19, 20). For example, IH has been shown to exacerbate metabolic dysfunction in obese patients, aggravating insulin resistance and nonalcoholic fatty liver disease (21). In individuals with metabolic syndrome, OSA is independently associated with higher blood glucose and triglyceride levels, as well as markers of inflammation, arterial stiffness, and atherosclerosis (21). Importantly, the effects of these mechanisms vary among individuals, forming the basis for the heterogeneity of OSA and its variable cardiovascular risk (2).

## 3 From traditional metrics to novel physiological indicators

For a long time, the apnea–hypopnea index (AHI) has been considered the gold standard for diagnosing OSA and assessing its severity. However, growing evidence indicates that the value of AHI as a predictor of cardiovascular risk is limited (5). AHI only accounts for the frequency of events, neglecting the duration of each respiratory episode, the severity of accompanying hypoxemia, and the resulting physiological compensatory responses, such as heart rate and blood pressure fluctuations. Consequently, two patients with the same AHI may experience entirely different hypoxic burdens and levels of cardiovascular stress.

**Figure 2:**
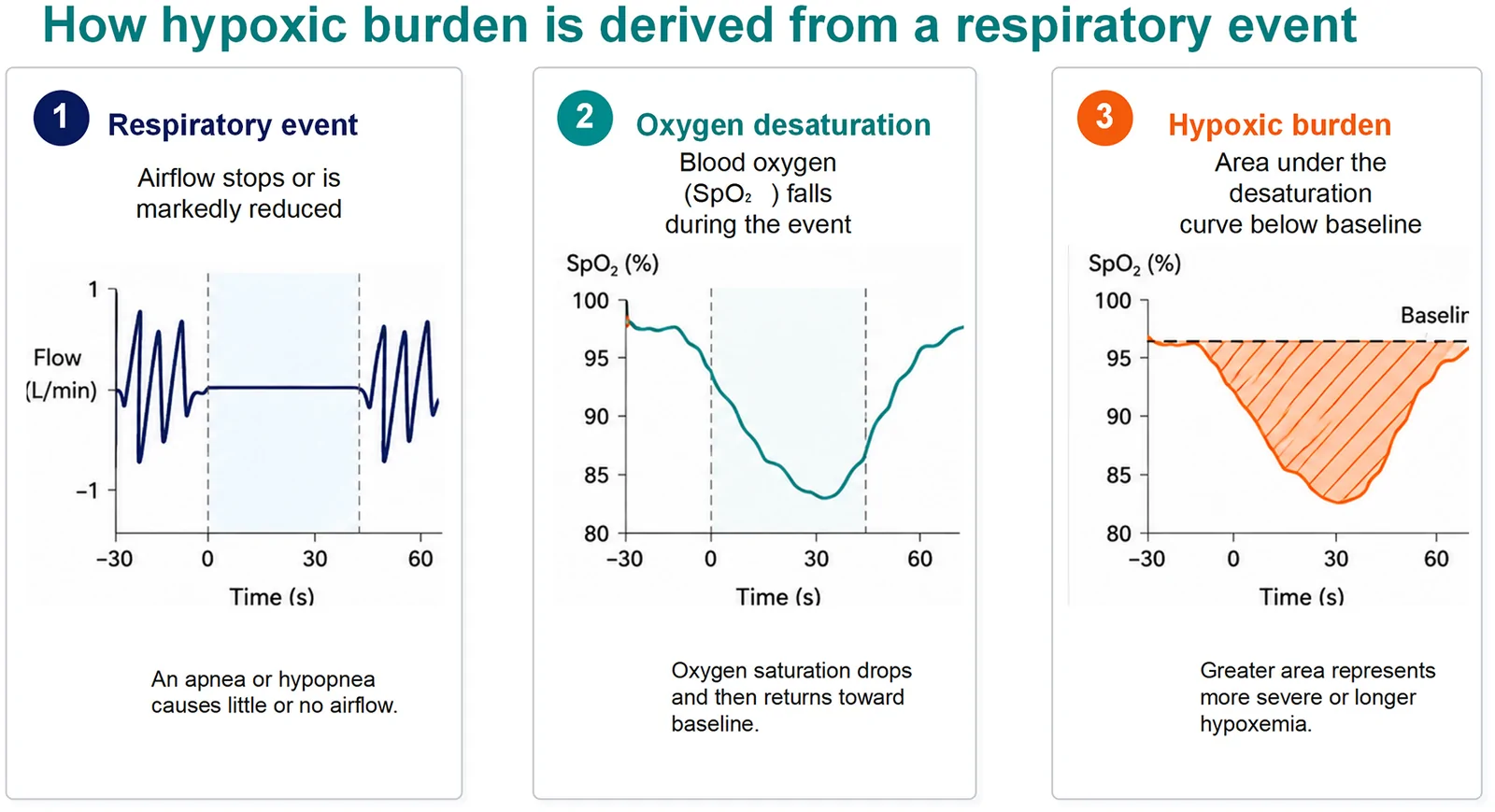
How hypoxic burden is derived from a respiratory event.

To more accurately assess cardiovascular risk in OSA, researchers have proposed a series of novel physiological indicators. Hypoxic burden (HB) is one such metric that has received considerable attention (Figure 2). It integrates both the depth and duration of oxygen desaturation during respiratory events, providing richer information on hypoxia than the AHI (22). Studies suggest that HB may have stronger correlations with endothelial dysfunction, subclinical atherosclerosis, and the risk of cardiovascular events (23). More importantly, reductions in HB resulting from treatment have been significantly associated with decreases in systolic blood pressure, indicating that HB could serve as a potential predictor of blood pressure response to therapy (24). Furthermore, indices related to hypoxic burden continue to be refined and expanded. Recently, a novel hypoxia burden index (HBI) was proposed, calculated by dividing the total desaturation area below 90% oxygen saturation by total sleep time, aiming to simultaneously capture the severity and duration of nocturnal hypoxia (25). In a retrospective study including 459 adults who underwent overnight polysomnography, HBI showed significant correlations with AHI, minimum oxygen saturation, and the proportion of sleep time spent below 90% oxygen saturation, while also exhibiting mild correlations with Epworth Sleepiness Scale scores, morning systolic and diastolic blood pressures, and mean arterial pressure. Notably, in a hospitalized patient subgroup, although patients with cardiovascular disease differed from those without in terms of age, sex, longest apnea duration, AHI, and HBI, multivariate analysis revealed that only age, sex, and HBI were independently associated with cardiovascular disease, whereas AHI showed no significant independent association. This finding further supports the potential value of hypoxic burden–related indices in cardiovascular risk assessment for OSA, suggesting that metrics integrating both the depth and duration of hypoxia may better reflect the risk of OSA-related cardiovascular injury than AHI alone.

Heart rate response is another promising metric. Fluctuations in heart rate during OSA events reflect the balance of the autonomic nervous system, particularly the degree of sympathetic activation. ΔHR has been shown to serve as a marker of cardiovascular risk in OSA patients. Patients with higher baseline ΔHR demonstrate more pronounced reductions in systolic blood pressure following CPAP or oxygen therapy (24). This suggests that ΔHR may help identify individuals whose cardiovascular systems are more sensitive to OSA-related stress and therefore more likely to benefit from treatment.

**Figure 3:**
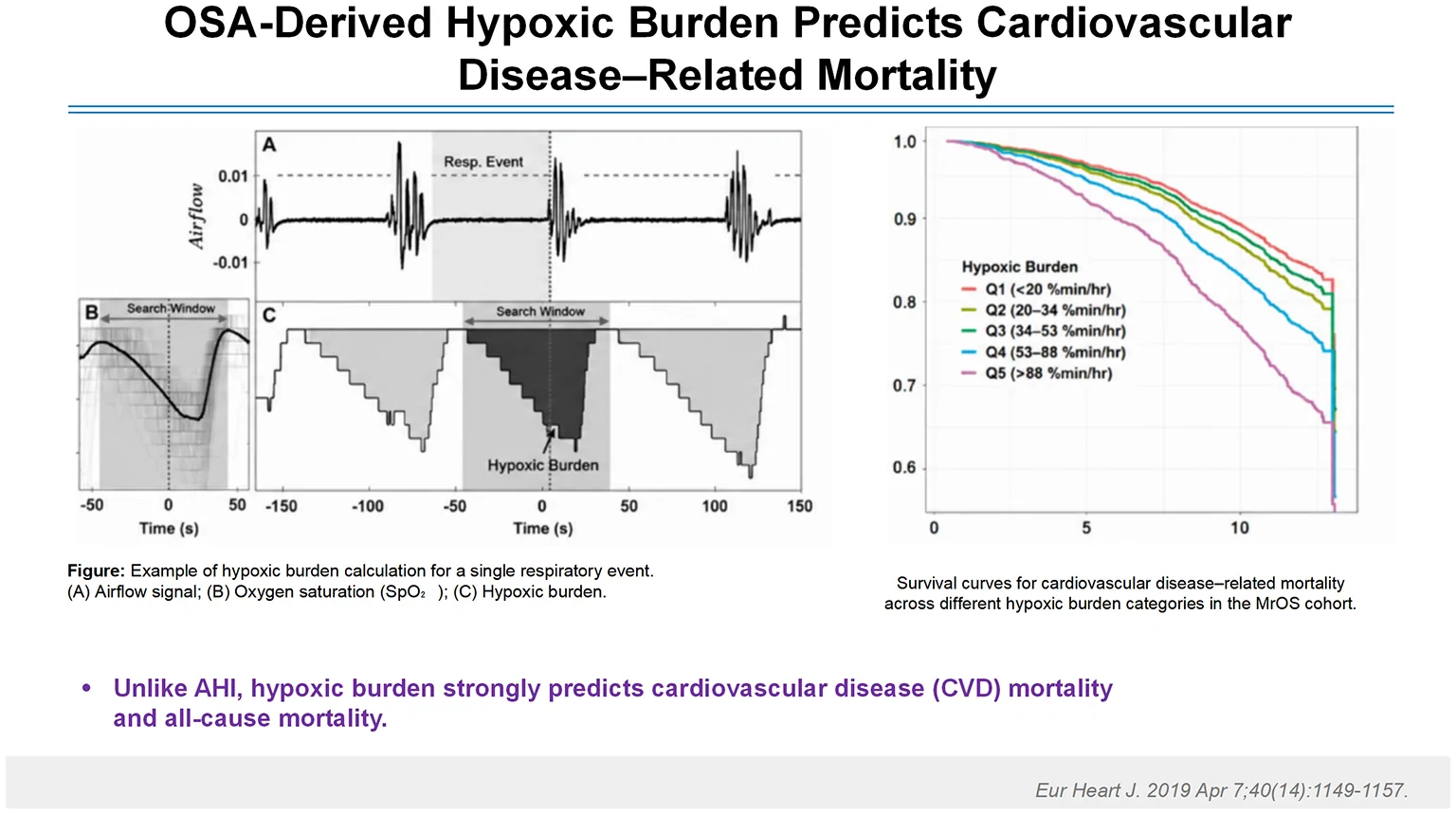
PPG and PWA assessment. PPG = photoplethysmography; PWA = pulse wave amplitude; PWAD = pulse wave amplitude drop. Reproduced from (29), Oxford University Press (**License Number** 6275921272891).

The pulse wave amplitude drop (PWAD) index is a recently emerging metric derived from photoplethysmography signals. PWAD reflects vascular constriction and reactivity induced by sympathetic activation. Studies have found that in OSA patients, lower PWAD indices—indicating impaired autonomic and vascular responsiveness—are independently associated with higher cardiovascular event risk (26) (Figure 3). Additionally, PWAD characteristics correlate with the Framingham 10-year cardiovascular risk score; among patients in sleep clinics with preexisting cardiovascular disease or elevated risk, PWAD indices are lower and PWAD durations are longer (27). These findings suggest that PWAD may serve as a biomarker of nocturnal vascular autonomic regulation, complementing existing risk assessment tools.

Moreover, metrics related to respiratory effort have also attracted attention. The percentage of respiratory effort time, measured via mandibular movements, has been shown to be a reliable predictor of hypertension in OSA patients, with predictive value surpassing that of traditional polysomnography parameters such as AHI and oxygen desaturation indices (28). A common feature of these novel physiological indicators is that they aim to quantify the actual “physiological burden” imposed by OSA events, rather than merely counting event frequency, providing the potential for more refined risk stratification (Table 1).

**Table 1:** Novel physiological metrics in OSA and their associations with cardiovascular outcomes.

Novel physiological metric	Calculation/Definition	Cardiovascular indicators and outcomes
Hypoxic burden	Calculated as the integral area of the oxygen desaturation curve during sleep divided by total sleep time (TST), yielding hypoxic burden per second (unit: % min/h).	Endothelial dysfunction, subclinical atherosclerosis, and blood pressure response to treatment
Hypoxia burden index	Calculated by dividing the total desaturation area of SpO_2_ below 90% by TST measured in seconds.	Morning blood pressure and prevalent cardiovascular disease (CVD)
Heart rate response	ΔHR is calculated as the subject–level mean of all differences between the maximal pulse rate following a respiratory event (within a subject–specific search window) and the nadir pulse rate during that respiratory event.	Blood pressure response to treatment
Pulse wave amplitude drop index (PWAD)	PWADs with an amplitude reduction of >30% from baseline and a duration of more than four heartbeats were identified. The total number of PWADs across the night was averaged per hour of sleep to derive the PWAD index (i.e., number of >30% drops in pulse wave amplitude per hour).	Risk of cardiovascular events
Respiratory effort–related metric	Percentage of time spent in respiratory effort as measured by mandibular movement.	Hypertension

## 4 Research frontiers in biomarkers

Circulating biomarkers hold significant potential in assessing cardiovascular risk in OSA due to their ease of collection and reproducibility. They can directly reflect downstream effects triggered by the pathophysiological mechanisms of OSA, including inflammation, oxidative stress, endothelial dysfunction, coagulation activation, and myocardial injury (Table 2).

**Table 2:** Blood biomarkers for cardiovascular risk stratification in obstructive sleep apnea.

Biomarker category	Representative biomarkers	Pathophysiological relevance in OSA	Cardiovascular indicators and outcomes
Metabolic endotoxemia–related markers	Lipopolysaccharide–binding protein (LBP)	LBP reflects metabolic endotoxemia. Its circulating levels increase with OSA severity and may indicate a mechanistic link between OSA, endotoxin exposure, and early vascular injury.	Increased carotid intima–media thickness
TNF receptor superfamily–related markers	Tumor necrosis factor receptor superfamily member 11B (TNFRSF11B/osteoprotegerin)	Associated with the presence and severity of OSA, potentially reflecting inflammatory vascular remodeling and endothelial dysfunction.	Endothelial dysfunction and vascular injury
Endothelial dysfunction markers	Endothelial cell–specific molecule−1 (ESM−1/endocan), ischemia–modified albumin (IMA)	ESM−1 is associated with OSA severity, particularly in patients with concomitant hypertension. IMA reflects ischemia–related albumin modification and is elevated in patients with acute coronary syndrome and OSA.	Hypertension, endothelial dysfunction, acute coronary syndrome, and recurrent cardiovascular events
Coagulation and platelet activation markers	Mean platelet volume (MPV), platelet aggregation	MPV is increased in OSA and positively correlated with disease severity, suggesting platelet activation. OSA severity is also associated with enhanced platelet aggregation among individuals receiving aspirin, suggesting possible attenuation of antiplatelet efficacy.	Cardiovascular disease risk, platelet hyperreactivity, thrombosis, and reduced aspirin responsiveness
Cardiac stress and myocardial injury markers	B–type natriuretic peptide (BNP), N–terminal pro–B–type natriuretic peptide (NT–proBNP), high–sensitivity cardiac troponin I (hs–cTnI)	BNP and NT–proBNP reflect myocardial wall stress, whereas hs–cTnI reflects subclinical myocardial injury. Some studies suggest associations with OSA severity, particularly in patients with cardiac involvement.	Heart failure risk, myocardial injury, diastolic dysfunction, and cardiac remodeling
Prognostic stress–response markers	Pentraxin-3	Pentraxin-3 is elevated in OSA patients with atrial fibrillation and may reflect systemic stress, inflammation, and adverse cardiovascular remodeling.	Atrial fibrillation and adverse cardiovascular prognosis
Circulating microRNAs	miR−107, miR−143, miR−486 and other OSA–related miRNA profiles	Circulating miRNAs regulate gene expression and may capture molecular signatures related to intermittent hypoxia, endothelial dysfunction, hypertension, and myocardial injury.	Hypertension, myocardial infarction, endothelial dysfunction, and cardiovascular risk scores

Inflammation and oxidative stress are central to OSA-induced cardiovascular damage. Multiple studies have confirmed that serum C-reactive protein (CRP) levels are elevated in OSA patients and independently correlate with severity indices such as AHI and oxygen desaturation index (30). In patients with acute ischemic stroke, moderate-to-severe OSA is significantly associated with elevated levels of tumor necrosis factor-alpha (TNF-α), interleukin-6 (IL-6), and plasminogen activator inhibitor-1 (13). Lipopolysaccharide-binding protein, a marker of metabolic endotoxemia, increases with OSA severity and independently predicts carotid intima-media thickness, suggesting that OSA may promote early atherosclerosis by exacerbating endotoxemia (31). Additionally, tumor necrosis factor receptor superfamily member 11B has been found to be independently associated with both the presence and severity of OSA, potentially contributing to endothelial dysfunction (32).

Endothelial dysfunction is an initiating factor in atherosclerosis. Beyond functional assessments using techniques such as peripheral arterial tonometry, circulating endothelial-specific molecules have become a focus of research. Endocan (endothelial cell-specific molecule-1, ESM-1) has been found to correlate with OSA severity, particularly in patients with concomitant hypertension, where serum ESM-1 levels serve as an independent predictor of OSA severity (33, 34). Another endothelial function marker, ischemia-modified albumin (IMA), is elevated in patients with acute coronary syndrome complicated by OSA; intriguingly, higher IMA levels appear to be associated with a lower risk of recurrent cardiovascular events (35).

Regarding coagulation abnormalities, mean platelet volume (MPV) is increased in OSA patients and positively correlates with disease severity, suggesting it may be one of the predictors of cardiovascular risk in this population (36). Additionally, studies have found that in individuals taking aspirin (primarily for primary prevention of cardiovascular disease), OSA severity is significantly associated with enhanced platelet aggregation, indicating that OSA may reduce the antiplatelet efficacy of aspirin or activate platelets through alternative pathways (37).

Biomarkers that directly reflect cardiac stress and injury have also attracted considerable attention. B-type natriuretic peptide (BNP) and N-terminal pro–B-type natriuretic peptide (NT-proBNP) are indicators of ventricular wall stress and heart failure, while high-sensitivity cardiac troponins (hs-cTn) reflect subtle myocardial injury. Although findings in OSA patients have been inconsistent, some studies have shown that these markers correlate with OSA severity, particularly in the presence of cardiac involvement (38, 39). For example, hs-cTnI levels are significantly higher in patients with severe OSA compared to those with mild-to-moderate disease and are associated with the degree of diastolic dysfunction (39). Pentraxin-3 is elevated in OSA patients with concomitant atrial fibrillation, suggesting a potential link to adverse outcomes (40).

In addition, microRNAs (miRNAs), as key regulators of gene expression, have shown potential as circulating biomarkers for cardiovascular risk stratification in OSA. Specific miRNA expression profiles have been associated with hypertension, myocardial infarction, or endothelial dysfunction in OSA patients. For instance, miR-107 and miR-143 are linked to particular blood pressure parameters in OSA patients, whereas miR-486 correlates with cardiovascular risk scores (41). These biomarkers, used individually or in combination, hold promise for identifying high-risk cardiovascular subgroups among OSA patients and guiding personalized therapy in the future.

## 5 OSA phenotypes and cardiovascular risk stratification

Recognizing the heterogeneity of OSA is key to achieving precise risk assessment and targeted therapy (2). OSA is not a homogeneous disease; rather, it comprises multiple subtypes resulting from different combinations of anatomical, physiological, inflammatory, and obesity-related risk factors, each characterized by distinct pathophysiological disturbances and clinical manifestations (2). Consequently, phenotype-based risk stratification strategies have emerged.

**Figure 4:**
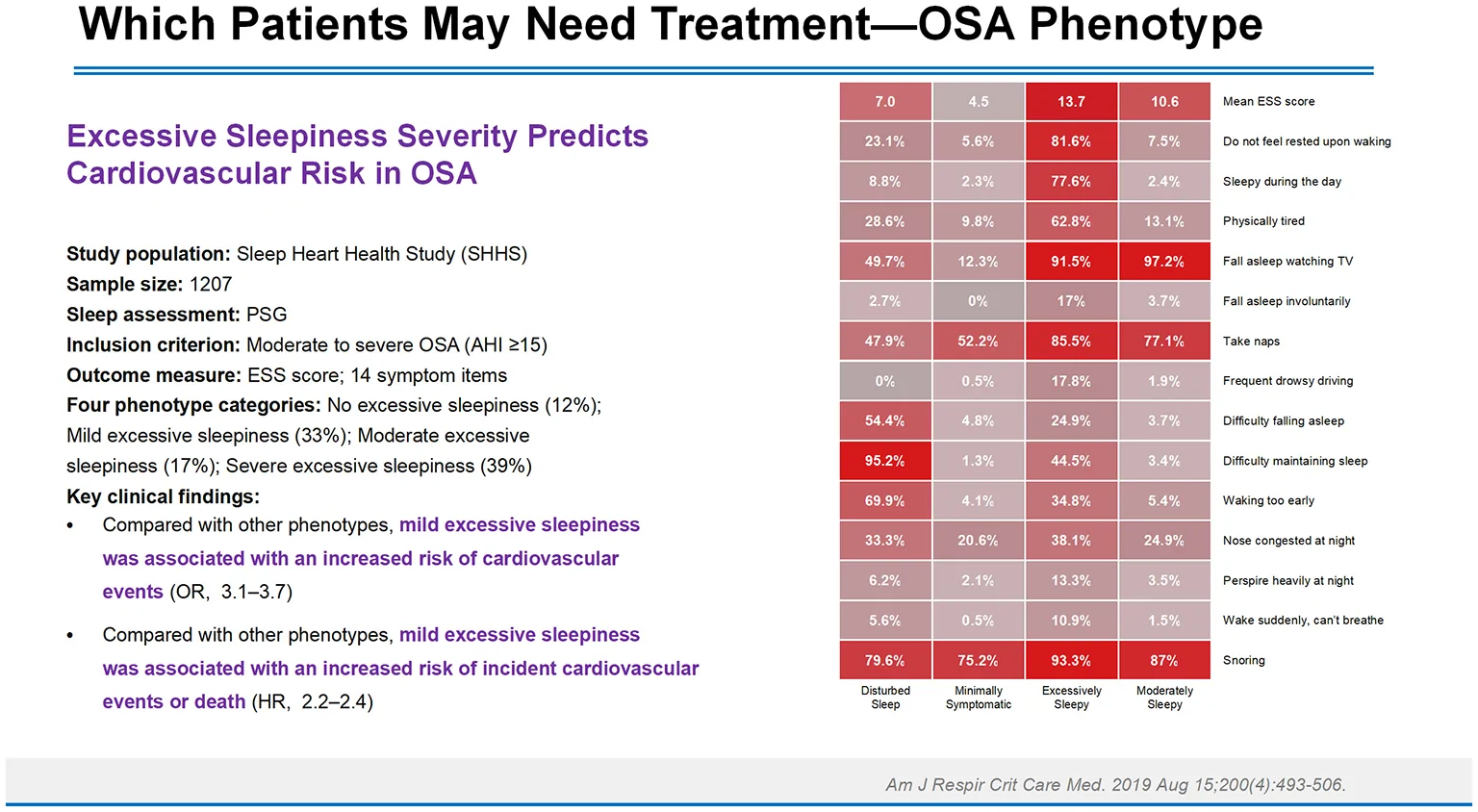
Excessive Sleepiness Phenotype in OSA and Cardiovascular Risk Prediction. Reproduced from (42), with permission from the American Thoracic Society. All rights reserved (**License Number** 6275961510168).

Symptom-based subtypes represent an important classification dimension. Traditional OSA diagnosis has focused on excessive daytime sleepiness, yet many patients do not exhibit obvious sleepiness. Studies indicate that OSA accompanied by daytime sleepiness may represent a phenotype with higher cardiovascular risk (42, 43) (Figure 4). In addition, the comorbidity of insomnia symptoms and OSA has increasingly gained attention. In particular, the insomnia subtype of OSA with objectively shortened sleep duration is associated with higher risks of hypertension, insulin resistance, and elevated inflammatory markers, suggesting that this subtype carries more pronounced cardiometabolic risk (44).

Hypoxic patterns represent another key basis for phenotypic differentiation. Cluster analyses based on event-specific hypoxic burden from apneas and hypopneas can identify OSA subtypes with distinct clinical characteristics. For example, studies have found that the “severe hypoxic burden with pronounced apnea-related desaturation” subtype exhibits a significantly higher prevalence of cardiometabolic syndrome and an elevated brain age index, a marker of cerebral aging, compared to other subtypes (45). This suggests that subtype classification based on hypoxic patterns may better capture pathophysiological heterogeneity relevant to clinical outcomes than reliance on AHI alone.

To integrate clinical symptoms, cardiovascular risk, and severity of respiratory disturbances, the Baveno classification and its modified version have been proposed to replace AHI as the sole criterion for treatment indication (46, 47). The modified Baveno classification incorporates cardiovascular risk assessment into the decision-making process, using symptomatology and cardiovascular risk to determine the intensity of treatment indication for patients with AHI < 30 events/hour. Retrospective analyses have shown that patients treated according to this classification experienced significant improvements in daytime sleepiness and office systolic blood pressure, with treatment prescription rates increasing in line with indication strength (46). A prospective multicenter study is currently underway to further validate the clinical utility of this classification (47). This multicomponent grading tool represents an important step toward individualized OSA management.

In addition, risk characteristics in specific populations require tailored consideration. For instance, the presentation and cardiovascular associations of OSA in women may differ from those in men (42). Among middle-aged women, moderate-to-severe OSA is independently associated with the presence of coronary artery calcification, suggesting that women are also susceptible to OSA-related cardiovascular consequences (48). In non-obese individuals and women, the association between OSA and intracranial arterial calcification appears more pronounced (49). These findings underscore the importance of considering factors such as sex and obesity status in cardiovascular risk stratification.

## 6 OSA risk assessment in the context of specific cardiovascular comorbidities

OSA frequently coexists with a variety of cardiovascular diseases, making risk assessment and management in these specific patient populations of significant clinical importance.

OSA is highly prevalent among patients with coronary artery disease (CAD). The prevalence of OSA is particularly high in patients with acute coronary syndrome (ACS) and is associated with adverse outcomes. OSA may influence the course of ACS by exacerbating plaque instability and promoting thrombosis. Studies have shown that in ACS patients with both high residual cholesterol and low-grade inflammatory risk, the presence of OSA significantly increases the risk of major adverse cardiovascular and cerebrovascular events, including stroke (50). Among patients undergoing percutaneous coronary intervention, OSA is an independent risk factor for cardiovascular events (51). Therefore, active screening for OSA is warranted in CAD patients, particularly those with ACS.

The relationship between OSA and heart failure is complex and bidirectional. OSA is a well-established risk factor for heart failure with reduced ejection fraction (HFrEF) and is also closely associated with heart failure with preserved ejection fraction (HFpEF) (52). HFpEF and OSA share many pathophysiological features, including systemic inflammation and oxidative stress; OSA may promote HFpEF development by inducing myocardial fibrosis and stiffness (52). The severity of OSA correlates with the degree of diastolic dysfunction and hs-cTnI levels, suggesting that it may contribute to repeated myocardial injury (39). In patients with established heart failure, treatment of OSA may help improve cardiac function.

OSA is a strong predictor of both the occurrence and recurrence of atrial fibrillation (AF) (53). The prevalence of AF is high among OSA patients (54). OSA may provide a substrate for AF through mechanisms such as atrial stretch, autonomic dysregulation, inflammation, and fibrosis (55). Studies have shown that OSA is strongly associated with cardioembolic stroke, a relationship that is independently of AF, suggesting the presence of undiagnosed paroxysmal AF or other embolic mechanisms (56). Among patients with newly diagnosed AF, the prevalence of OSA and the proportion requiring CPAP therapy are high, making screening with tools such as the STOP-Bang questionnaire a feasible strategy (57).

In the field of cerebrovascular disease, OSA is an independent risk factor for ischemic stroke (19). The prevalence of sleep-disordered breathing is extremely high among cerebrovascular disease patients, with over 60% exhibiting OSA (58). OSA may increase stroke risk through multiple pathways, including the induction of hypertension, promotion of atherosclerosis, creation of a hypercoagulable state, and triggering of AF (59). Among patients with carotid artery stenosis, the prevalence of OSA can reach up to 80% (60). Furthermore, OSA severity is independently associated with the degree of intracranial arterial calcification, particularly in women and non-obese individuals (49). Therefore, assessment and management of OSA are crucial in both primary and secondary stroke prevention.

## 7 Clinical challenges and future directions in cardiovascular risk assessment for OSA

Despite significant progress in cardiovascular risk assessment for OSA, the clinical application of these advances still faces numerous challenges, and future research must address these unmet needs.

First, integrating novel risk assessment indicators into clinical decision-making remains a major challenge. AHI is deeply entrenched as the diagnostic standard, while metrics such as hypoxic burden (HB) and pulse wave amplitude drop (PWAD) are largely still in the research phase, lacking standardized measurement protocols, defined normal ranges, and clear intervention thresholds (61). Integrated tools such as the modified Baveno classification provide a framework, but their effectiveness and feasibility in broader populations require validation through prospective studies (47). Future guidelines need to clarify when and how these novel indicators should be used to supplement or partially replace traditional assessments.

Second, predicting treatment response is central to precision medicine. Some negative results from large randomized controlled trials may be attributable to the failure to identify subgroups most likely to benefit from therapy (2). Indicators such as ΔHR and HB have shown potential in predicting blood pressure response to treatment (24). Additionally, studies indicate that patients with very severe OSA continue to experience significantly higher rates of cardiovascular events even with CPAP therapy, suggesting that this population may require more intensive or combination systemic treatments (62). Future clinical trials should adopt enrichment designs, enrolling patients with high-risk physiological features (e.g., elevated ΔHR, low PWAD indices) to more effectively evaluate the impact of interventions on cardiovascular outcomes.

Third, the clinical translation pathways for specific biomarkers remain unclear. Although numerous biomarkers are associated with OSA and its severity, their specificity, sensitivity, and cost-effectiveness as screening or monitoring tools in routine clinical practice require further evaluation (30). For example, circulating miRNA profiles show promise, but issues related to stability, assay standardization, and result interpretation remain barriers. Combining biomarkers with physiological indicators and clinical features to construct multimodal risk prediction models may represent a more feasible approach.

Moreover, cardiovascular risk assessment in the context of comorbidities is more complex. When comorbid conditions such as diabetes, obesity, or chronic kidney disease are present, it is difficult to isolate the specific contribution of OSA to cardiovascular risk (63, 64). For instance, in patients with type 2 diabetes, OSA severity is not associated with microvascular endothelial dysfunction but remains correlated with elevated blood pressure and reduced health-related quality of life (63). Future research should clarify which OSA-related indicators best indicate additional cardiovascular risk across different comorbid backgrounds.

Finally, treatment adherence continues to be a key confounding factor affecting outcome evaluation. Most studies confirm that only good CPAP adherence—typically defined as ≥4 h of use per night—yields cardiovascular benefits (1, 65). Therefore, future risk stratification models should also consider predicting patient adherence or explore effective alternative therapies with lower adherence requirements.

## 8 Conclusion and perspectives

OSA is a heterogeneous disorder closely linked to cardiovascular disease. Traditional metrics such as the AHI have clear limitations in predicting cardiovascular risk, as they fail to fully capture the pathophysiological complexity and inter-individual variability of the disease. In recent years, research has focused on developing novel indicators that better quantify the actual physiological burden of OSA. Physiological metrics such as hypoxic burden, heart rate response, and PWAD index, alongside circulating biomarkers reflecting inflammation, endothelial dysfunction, coagulation, and myocardial injury, provide powerful tools for more refined cardiovascular risk assessment.

Phenotype-based stratification strategies integrating symptoms, hypoxic patterns, and cardiovascular risk—such as the Baveno classification—represent an important step toward individualized OSA management. In patients with specific cardiovascular comorbidities, including coronary artery disease, heart failure, atrial fibrillation, and stroke, screening and risk assessment for OSA hold particular clinical significance, as they directly influence disease progression and prognosis.

Looking ahead, research on cardiovascular risk assessment in OSA should focus on several directions. First, standardizing and clinically validating novel physiological metrics and biomarkers, and establishing their clear associations with hard cardiovascular outcomes. Second, designing and conducting prospective, enriched-population intervention trials to determine whether targeted therapy guided by novel risk stratification can improve cardiovascular outcomes. Third, leveraging artificial intelligence and machine learning to integrate multidimensional data—including polysomnography parameters, biomarkers, imaging, and clinical symptoms—to construct more robust risk prediction models. Fourth, exploring pharmacological interventions targeting specific pathophysiological pathways, such as inflammation or sympathetic activation, as adjuncts or alternatives to CPAP, particularly for patients with poor adherence or high risk.

The ultimate goal is to achieve precision management of OSA: accurately identifying patients for whom OSA is a major driver of cardiovascular risk and providing the most effective interventions, thereby truly reducing the cardiovascular disease burden in this large population.
